# Comparative transcriptome analysis of *longissimus dorsi* tissues with different intramuscular fat contents from Guangling donkeys

**DOI:** 10.1186/s12864-022-08857-2

**Published:** 2022-09-09

**Authors:** Wufeng Li, Lixia Qiu, Jiawei Guan, Yutong Sun, Jingwei Zhao, Min Du

**Affiliations:** 1 College of Life Sciences, Shanxi Agricultural University, Taigu, 030801 People’s Republic of China; 2 Department of Animal Sciences, Washington Center for Muscle Biology, Washington State University, Pullman, Washington, 99164-6310 USA

**Keywords:** Guangling donkeys, IMF, Transcriptome, DEGs, Lipid deposition

## Abstract

**Background:**

Donkey meat has low fat and high protein contents and is rich in various unsaturated fatty acids and trace elements that are beneficial to human digestion and absorption. IMF (intramuscular fat), also known as marbling, is an important indicator of the lean meat to fat ratio, which directly affects the tenderness and juiciness of the meat. At present, the underlying molecular variations affecting IMF content among donkey breeds are unclear. The Guangling donkey is an indigenous species in China. This study explored candidate regulatory genes that affect IMF content in Guangling donkeys. The IMF content of the *longissimus dorsi* muscle in 30 Guangling donkeys was measured. Six donkeys of similar age were selected according to age factors and divided into two groups, the high (H) and low (L) fat groups, according to their IMF content.

**Results:**

RNA-seq technology was used to compare the muscle transcriptome between the two groups. More than 75.0% of alternative splicing (AS) events were of the skipped exon (SE) type. A total of 887 novel genes were identified; only 386 novel genes were aligned to the annotation information of various databases. Transcriptomics analysis revealed 167 differentially expressed genes (DEGs), of which 64 were upregulated and 103 were downregulated between the H and L groups. Gene ontology analysis showed that the DEGs were enriched in multiple biological processes and pathways that are related to adipocyte differentiation, lipid synthesis, and neutral lipid metabolism. KEGG pathway analysis suggested that arachidonic acid metabolism, the HIF-1 signalling pathway, fructose and mannose metabolism, glycerophospholipid metabolism, and the AMPK signalling pathway were involved in lipid deposition. In addition, a gene–gene interaction network was constructed that revealed that the DEGs, including *SCD*, *LEPR*, *CIDEA*, *DLK1*, *DGAT2*, *ITGAL*, *HMOX1*, *WNT10B*, and *DGKA,* had significant roles in adipocyte differentiation and adipogenesis. The selected DEGs were further validated by qRT–PCR.

**Conclusion:**

This study improves the in-depth understanding of gene regulation and protein expression regarding IMF deposition and lays a basis for subsequent molecular breeding studies in Guangling donkeys.

**Supplementary Information:**

The online version contains supplementary material available at 10.1186/s12864-022-08857-2.

## Background

Guangling donkeys are distributed in Guangling County, Shanxi Province, China, and they are a local dominant breed that is carefully reared by local people using traditional production practices [1]. Guangling donkeys have a stout physique and full muscles. Thus, there is a huge market demand for the high-yield of their quality meat with special nutritional value  (Zhang et al. [2]). Guangling donkeys used for meat production have a high intramuscular fat (IMF) content; however, the underlying molecular mechanisms underlying the IMF variation among donkey species are not fully understood [3]. IMF, also known as marbling, is the fat tissue that accumulates between muscle fibres and primary muscle bundles. This affects the tenderness, juiciness, and flavour of the meat [4]. Therefore, IMF significantly affects the consumer’s sensory judgement and quality evaluation of meat. Meat with a low IMF content is dry and has poor flavour. IMF content is one of the most important indicators used to evaluate meat quality [5]. IMF content is a polygenic trait that is regulated by many genes affecting adipogenesis and fat metabolism [6]. Many factors, including sex, age, breed, nutrition, and genetics, can significantly affect muscle IMF deposition [7]. However, the IMF content can only be measured after animal slaughter, which cannot be used for traditional breeding selection based on individual phenotypes and pedigrees. The existing ultrasonic method can be used to detect the IMF content in slaughtered meat samples but cannot be widely used in different species, especially in donkeys. Additionally, there are only a few related research reports on the use of this method, so the accuracy of the method is not certain. Therefore, to overcome the limitations of conventional IMF content selection practices, identifying molecular markers for genetic selection is important.

Next-generation sequencing (NGS) for transcriptome analysis (RNA-seq) has been widely used to examine complex traits, including fat deposition. Using RNA-seq technology, a total of 578 differentially expressed genes (DEGs) were identified in the *longissimus dorsi* and visceral adipose tissue (VAT) of Dezhou donkeys. Compared with VAT, 267 genes were upregulated and 311 genes were downregulated in the *longissimus thoracis*. KEGG enrichment analysis revealed that glycerolipid and glycerophospholipid metabolism are key metabolic pathways regulating lipid deposition [8]. In another study, to compare IMF gene expression patterns, differential expression analysis was performed on the coexpressed mRNAs and the differentially expressed mRNAs in the muscle of Xinyang buffalo and Nanyang cattle. In total, 1566 differentially coexpressed mRNAs and 70 differentially expressed muscle-specific mRNAs were identified. PCK1 was found to be more abundant in adipose tissue than in muscle tissue. In muscles, the expression of PCK1 in Nanyang cattle was found to be higher than that in water buffalo, indicating the positive role of PCK1 in IMF deposition in cattle [9]. Although there have been some transcriptome studies in donkeys and cattle exploring the mechanisms underlying differences in IMF contents, these studies failed to fully describe the mechanism underlying IMF variation. In particular, the mechanisms underlying IMF deposition in Guangling donkeys remain unclear.

This study used RNA-seq for transcriptome analysis of *longissimus dorsi* tissues to assess different IMF contents among Guangling donkeys by analysing the DEGs and their associated pathways. The findings provide a theoretical basis for the genetic breeding of Guangling donkeys.

## Results

### Measurement and analysis of IMF in Guangling donkeys

The IMF content of the *longissimus dorsi* in 30 Guangling donkeys varied from 2.23 to 9.00%. The 6 selected donkeys (three in each group) with the lowest and highest IMF contents were named the L group and H group, respectively. Then, RNA-seq was performed. The individual samples in the groups were named L1, L2, L3, H1, H2, and H3 (Table 1).

**Table 1:** Descriptive statistics of female donkeys

	L (*n* = 3)	H (*n* = 3)	*P* value	R squared
IMF content±SEM	2.45 ± 0.14	8.88 ± 0.09	< 0.0001	0.9975

### Statistics and evaluation of sequencing data

The *longissimus dorsi* transcriptomes of Guangling donkeys were sequenced using the Illumina NovaSeq sequencing system I. More than 57.6 million raw reads were obtained from each group (Table 2). The data were screened to remove low-feasibility and poly-N-containing reads. More than 56.45 million clean reads were obtained for subsequent analysis. Approximately 95% of the obtained genes were successfully matched to the *Equus asinus* reference genome. The Q30 scores in each group were > 93%.

**Table 2:** Statistics of the quality and alignment efficiency of transcriptome sequencing

Sample	Raw Reads	Clean Reads (%)	Reads mapped (%)	Mapped to exon (%)	Clean Base (G)	Q30 (%)
H1	57,607,030	56,456,182 (98.00)	53,525,206 (94.81%)	78.70	8.47	93.8
H2	67,485,908	66,252,450 (98.17)	63,065,876 (95.19%)	85.88	9.94	95.36
H3	59,360,730	58,221,334 (98.08)	55,414,202 (95.18%)	87.85	8.73	95.22
L1	67,336,812	66,247,312 (98.38)	62,894,562 (94.94%)	83.57	9.94	94.94
L2	64,402,184	63,211,670 (98.15)	60,222,790 (95.27%)	87.50	9.48	95.24
L3	62,628,782	61,565,326 (98.30)	58,822,760 (95.55%)	86.58	9.23	95.55

Q30: A percentage of the bases with a Qphred value of not less than 30

Reads mapped: Number of reads mapped to the reference genome

### DEGs

**Fig. 1:**
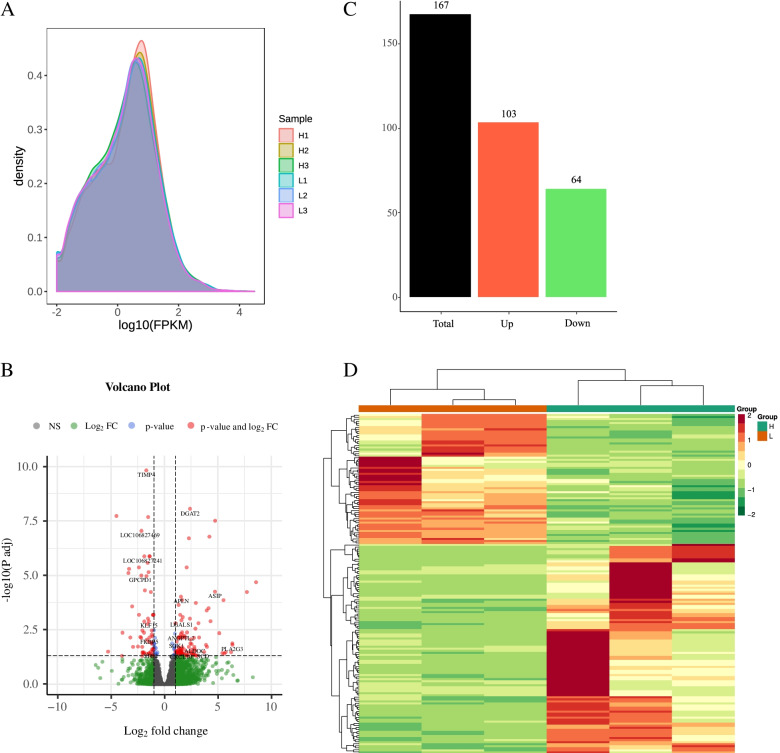
Differential expression analysis of genes between the high (H) and low (L) IMF content groups (**A**). Density plot of gene expression density distribution in each sample (**B**). Volcano plot of DEGs between the H and L IMF groups (**C**). The X-axis and Y-axis represent the values of log2 (H/L) and –log10 (p-adj), respectively. Distribution map of upregulated and downregulated genes in the H and L IMF groups (**D**). Heatmap of DEGs among the six samples

To screen out candidate genes that are significantly associated with changes in the IMF content in donkeys, the DEGs between the L and H groups were obtained using DESeq2. Notably, the gene density distribution between the H and L groups was almost the same without a significant difference (Fig. 1A). However, 167 DEGs were identified based on the criteria |log_2_ Fold-Change | > = 1 and FDR < 0.05. In the H group, 103 genes were upregulated (61.67%), and 64 genes were downregulated (38.32%) (Supplementary Table S1, Fig. 1B and C). For example, the *SCD* (log_2_ Fold-Change = 3.718), *DGAT2* (log_2_ Fold-Change = 2.394), and *CIDEA* (log_2_ Fold-Change = 2.702) genes were differentially expressed and are considered to be associated with a high IMF content. Figure 1D shows the heatmap profile of the DEGs in the H and L samples.

### Functional enrichment analysis of DEGs

**Fig. 2:**
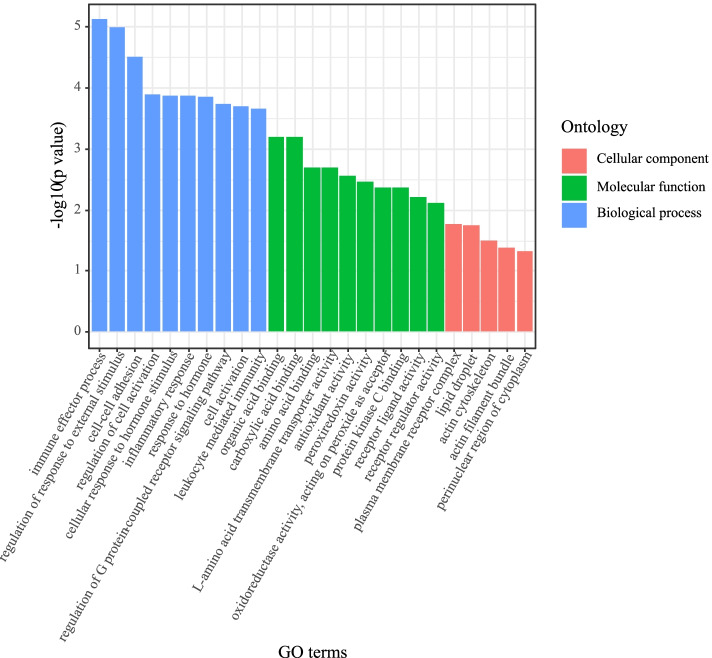
GO enrichment analysis of the DEGs between the high (H) and low (L) IMF content groups. The Y-axis represents the -log10 value (*p* value), and the abscissa represents the GO terms

Next, GO functional enrichment analysis was performed to explore the functions of the DEGs. GO terms included three categories: cellular composition, molecular function, and biological processes (Supplementary Table S2, Fig. 2). The results showed that 400 GO terms were significantly enriched in the three categories (*P* < 0.05). The biological processes included intercellular adhesion (GO: 0098609), the regulation of fat cell differentiation (GO: 0045598), smooth muscle cell proliferation, negative regulation (GO: 0048662), cellular response to lipids (GO: 0071396), the neutral lipid metabolic process (GO: 0006638), the neutral lipid biosynthetic process neutral lipid biosynthesis (GO: 0046460), and the negative regulation of MAPK cascade (GO: 0043409). The molecular functions included protein kinase C binding (GO: 0005080), carboxylic acid binding (GO: 0031406), organic acid binding (GO: 0043177), the triglyceride biosynthetic process (GO: 0019432), the acylglycerol biosynthetic process (GO: 0046463), the response to fatty acids (GO: 0070542), and the acylglycerol metabolic process (GO: 0006639). The cellular composition included lipid droplets (GO: 0005811) and actin cytoskeleton (GO: 0015629). These data showed that the DEGs are closely related to cell development and fat metabolism.

### KEGG analysis of the DEGs

**Fig. 3:**
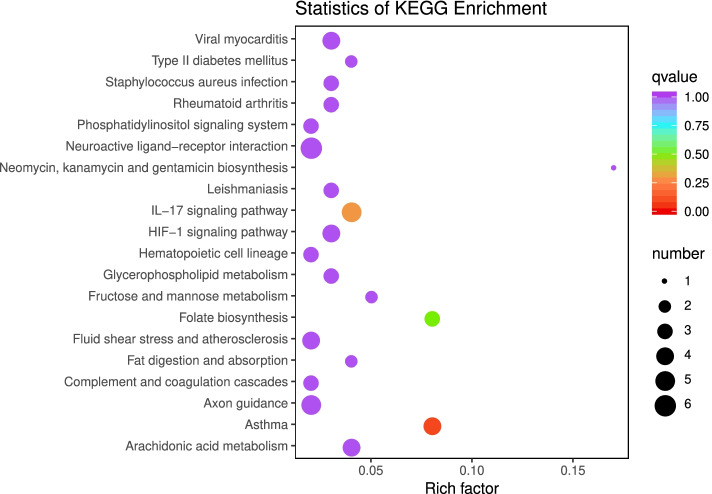
Scatter plot showing the enrichment analysis of the top 20 KEGG pathways. A larger Rich factor indicates higher enrichment. Larger points indicate a higher number of DEGs enriched in that pathway

Next, KEGG enrichment analysis revealed that a total of 177 metabolic pathways were enriched in the DEGs. The top 20 enriched KEGG metabolic pathways are shown in Fig. 3. Among them, 12 KEGG pathways were found to be significantly enriched (*P* < 0.05), such as arachidonic acid metabolism, the HIF-1 signalling pathway, fructose and mannose metabolism, glycerophospholipid metabolism, and the AMPK signalling pathway (Table 3). This suggests that genes involved in glucose and lipid metabolism pathways might be candidate genes affecting the IMF content of meat.

**Table 3:** The partially significantly enriched KEGG pathways between the high (H) and low (L) IMF content groups

KEGG Pathway	KEGG Pathway ID	Number and ratio of DGEs	Gene name
IL-17 signalling pathway	ko04657	5 (6.49%)	*TRAF3*; *IL17B*; *MAPK4*; *CXCL10*; *NTSR2*
Arachidonic acid metabolism	ko00590	4 (5.19%)	*PLA2G3*; LOC106827241; LOC106827469; *CYP4F*
HIF-1 signalling pathway	ko04066	4 (5.19%)	*HMOX1*; *HK2*; *ALDOC*; *FLT1*
Fructose and mannose metabolism	ko00051	2 (2.59%)	*HK2*; *ALDOC*
Glycerophospholipid metabolism	ko00564	3 (3.89%)	*DGKA*; *PLA2G3*; *GPCPD1*
AMPK signalling pathway	ko04152	3 (3.89%)	*SCD*; *LEPE*; *PP2A*

### Interaction network of DEGs

**Fig. 4:**
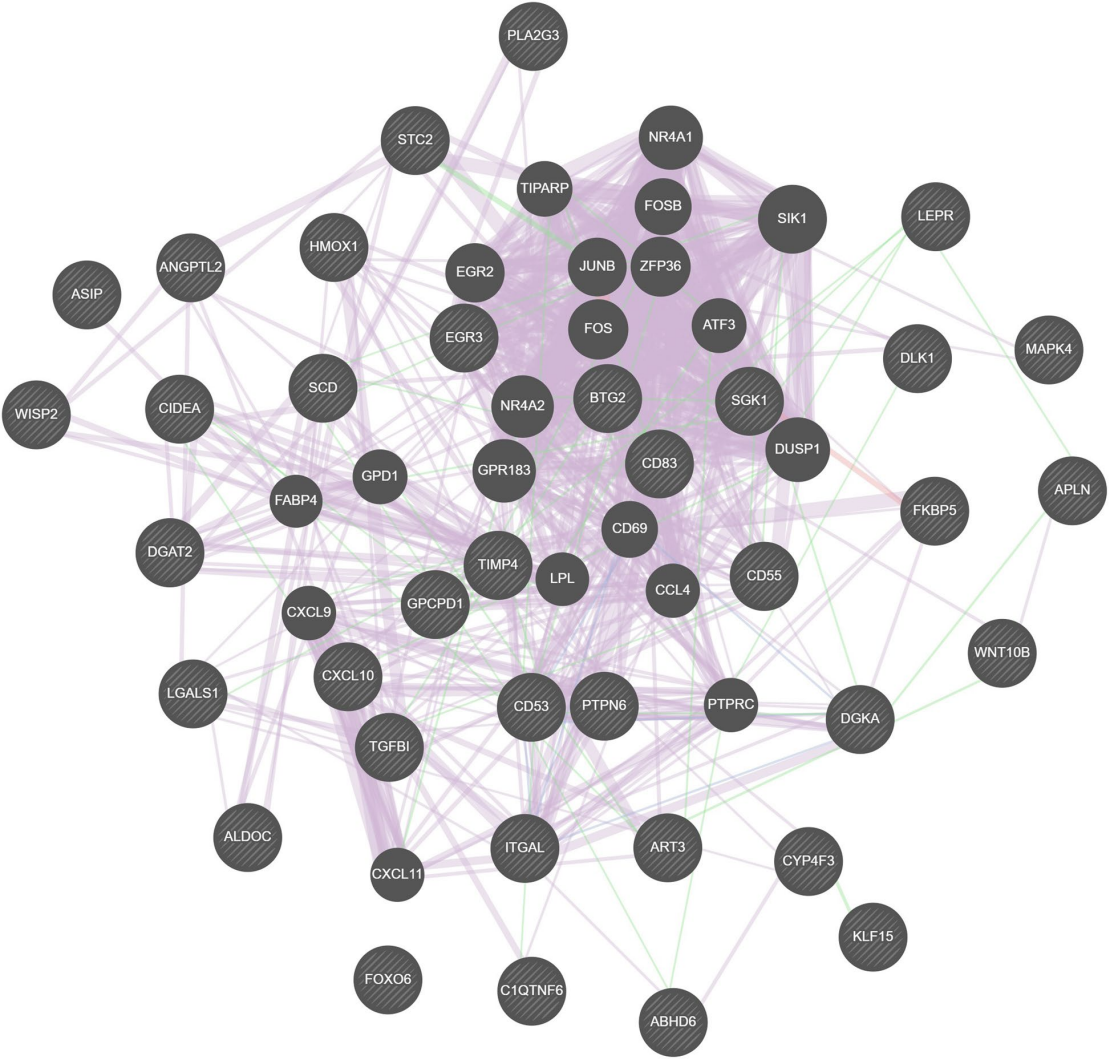
The coexpression network of the DEGs constructed by GeneMANIA

**Fig. 5:**
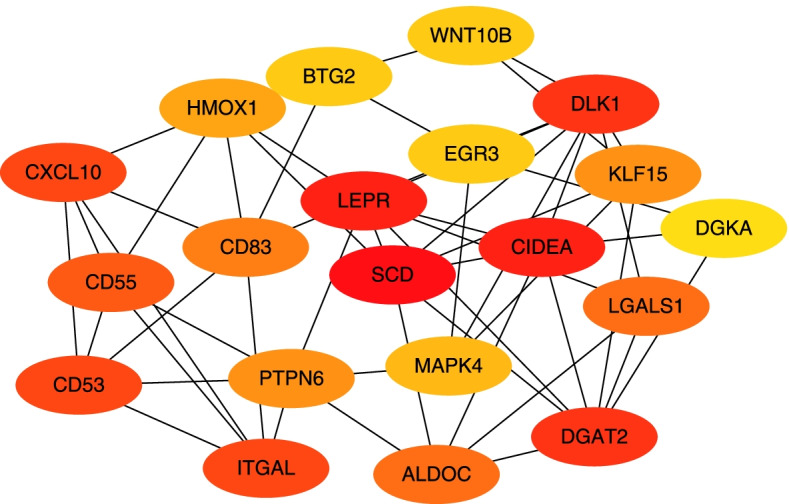
Subnetwork of the core DEGs with ≥4.0 degrees and |log_2_ Fold-Change| > =1 (32 nodes and 76 edges). The colour intensity shows the ranking position. The dark red genes have the highest maximum group concentration (MCC) value, suggesting higher importance in the network. Correspondingly, light yellow has a lower MCC value, suggesting lower importance

This study mainly focused on the fat metabolism pathway, as a large number of DEGs (38 genes) were related to fat metabolism (Supplementary Table S3). Therefore, a coexpression network was constructed for these 38 DEGs (Fig. 4). A total of 1079 interactions were observed. The genes with the highest network weights included *ITGAL*, *TIMP4*, *SCD*, *DGAT2*, *DGKA*, *HMOX1*, and *CIDEA*, which may play important roles in IMF deposition. Another interaction network was constructed to analyse the coexpressed genes with ≥4.0 degrees (Fig. 5). Based on maximum clique centrality (MCC) scores, the most important gene nodes included *SCD*, *LEPR*, *CIDEA*, *DLK1*, *DGAT2*, *ITGAL*, *HMOX1*, *WNT10B*, and *DGKA*.

### qRT–PCR validation of DEGs

**Fig. 6:**
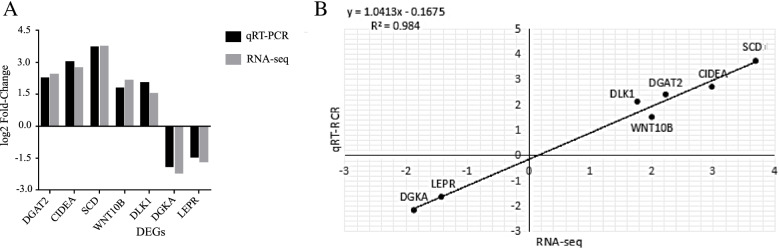
qRT–PCR validation of 7 DEGs (**A**). The x-axis shows the names of 7 DEGs, and the y-axis represents the corresponding log_2_ fold-change derived from RNA-seq and qRT–PCR (**B**). Regression analysis between qRT–PCR (x-axis) and RNA-seq (y-axis) data was performed according to the log_2_ fold-change value. The regression equation is y = 1.0413x-0.1675, and R^2^ = 0.984. The black dots indicate the correlation data of the 7 DEGs

To validate the RNA-seq data, seven DEGs (*DGAT2*, *SCD*, *LEPR*, *DLK1*, *WNT10B*, *CIDEA*, and *DGKA*) were randomly selected and confirmed by qRT–PCR. The results showed a similar trend with a correlation of 0.992 (R^2^ = 0.984), indicating the reliability of the RNA-seq results (Fig. 6A and B).

### Novel genes and the prediction of AS

**Fig. 7:**
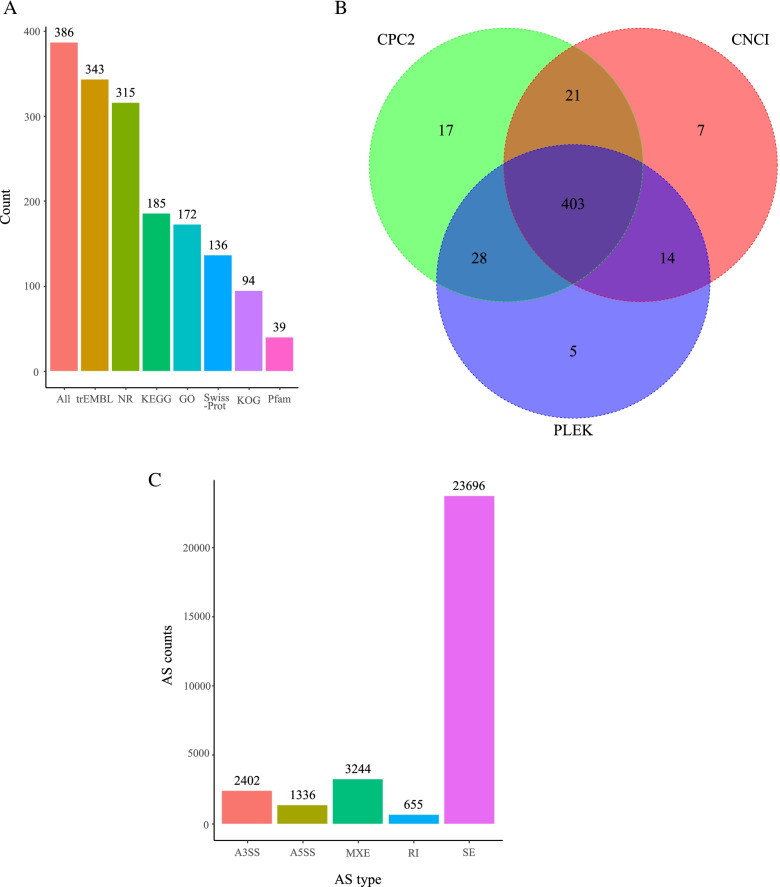
The number of novel genes in various databases (**A**). The novel genes and predicted AS sites (**B**). The predicted number of AS sites and AS types in the *longissimus dorsi*

A total of 887 novel genes were obtained after comparing the spliced transcripts with the genome annotation data, and only 386 genes were aligned with the annotation information in the databases. (Fig. 7A). The coding potential of 887 novel genes was predicted by software, and 484 mRNAs and 403 lncRNAs and their corresponding starting positions were obtained (Supplementary Table S4, Fig. 7B). In total, 31,333 AS sites were identified (Fig. 7C) in the *longissimus dorsi* of the Guangling donkey, and these included all 5 types of AS. The skipped exon (SE) type accounted for 75% of AS sites, indicating its higher presence in the *longissimus dorsi* of the Guangling donkey. These results provide a good reference for AS events in donkey muscle as they relate to IMF deposition.

## Discussion

Fat deposition is a dynamic accumulation process that results from a higher energy intake than required [10]. Commitment and terminal differentiation are the two main stages of adipogenesis. During the commitment stage, mesenchymal stem cells (MSCs) are converted to committed white preadipocytes. The expression of the marker genes *PPARγ* and *C/EBPs* induces adipocyte differentiation. During terminal differentiation, certain genes involved in the synthesis and degradation of triacylglycerol, such as the *DGAT2* gene, are upregulated, which converts progenitor cells into mature white adipocytes [11]. Advanced high-throughput sequencing technology can analyse the differences between high- and low-fat samples to explore the mechanisms underlying IMF deposition in donkeys. For instance, to explore the expression and function of circRNAs related to IMF content in donkeys, a high-throughput sequencing study identified 127 differentially expressed (DE) circRNAs in the high and low IMF groups of Liaoning donkeys and further predicted and analysed 127 DEcircRNAs that may interact with miRNAs [12]. Notably, multiple circRNAs may act as miRNA sponges regulating adipocyte differentiation. Based on the reference genome version used, 887 novel genes were detected in the transcriptome results of this study. The gene sequences whose class codes were annotated by “u”, length > 200 nt, and exon number > 1 were retained and subjected to novel gene annotations. The transcriptome analysis of Dezhou donkeys detected 6290 novel genes in one study [13]. These results differed from our results, largely due to different versions of the reference genome. More importantly, we found 167 DEGs, including 64 upregulated and 103 downregulated genes.

GO enrichment analysis indicated that many DEGs were enriched in biological pathways related to the regulation of fat cell differentiation, lipid biosynthesis, and metabolic processes. This indicated that there was a significant difference in lipid metabolism between the L and H groups. Moreover, we must admit that there were changes in the IMF contents of the Guangling donkeys aged between 24 and 36 months, and therefore, age, may be a significant factor. To obtain accurate inferences about IMF content, more follow-up experiments are needed in the future. Furthermore, KEGG pathway enrichment analysis revealed that the DEGs were significantly enriched in arachidonic acid metabolism, glycerophospholipid metabolism, fat digestion and absorption, and the AMPK signalling pathway. Among them, arachidonic acid metabolism and the AMPK signalling pathway play important roles in fat metabolism. Arachidonic acid (AA), a polyunsaturated fatty acid, is a precursor for the synthesis of bioactive mediators and plays a complex role in fat synthesis [14]. AMPK signalling is a key pathway that regulates energy metabolism by reducing anabolism involving synthetic fat genes, ribosomal protein translation, cholesterol, and fatty acid synthesis and enhancing catabolism involving glucose and fat transport, fatty acid oxidation, and oxidative metabolism to increase the intracellular ATP reserve [15]. Activated AMPK promotes the oxidative breakdown of lipids and inhibits glycogen and lipid synthesis [16, 17]. These data suggest that the DEGs involved in energy metabolism and fatty acid synthesis might be responsible for the different IMF deposition between the L and H groups. Furthermore, a gene-gene interaction network was constructed and identified genes, such as the *SCD*, *LEPR*, *CIDEA*, *DLK1*, *DGAT2*, *ITGAL*, *HMOX1*, *WNT10B*, and *DGKA*, that might have critical roles in IMF deposition.

Compared with the L group, the H group had more genes related to the first phase of adipocyte differentiation; among those, *WNT10B* and *DLK1* were upregulated. The Wnt signalling pathway is closely related to adipocyte development and differentiation. The *WNT10B* gene, a member of the *WNT* gene family, inhibits adipogenesis by inhibiting key transcription factors, such as *C/EBPα* and *PPARγ* [18]. Interestingly, the high expression of *WNT10B* in the H group suggests that maintaining a balance between anabolic and catabolic lipid metabolism is highly complex. *DLK1* (delta-like noncanonical Notch ligand 1), also known as preadipocyte factor l (*pref-l*), serves as a marker for preadipocytes. The overexpression of *Pref-1* inhibits adipogenic differentiation through the downregulation of *PPARγ* and *C/EBPα* [19, 20]. However, some studies have shown that the *DLK1* gene may enhance the differentiation of adipocytes and is positively correlated with fat content [21, 22]. The expression of haem oxygenase-1 (encoded by the *HMOX1* gene) was downregulated in the H group; haem oxygenase-1 is a widely expressed microsomal enzyme in mammals, including humans. *HMOX1* exerts multiple effects, such as anti-inflammatory, anti-apoptotic, and antioxidant properties, through multiple signalling pathways [23]. *HMOX1* can upregulate the Wnt/β-catenin signalling pathway, which inhibits the activities of *PPARγ* and *C/EBPa*. In turn, adipogenesis is reduced [24, 25]. Similarly, *HMOX1* overexpression inhibits mesenchymal stem cell differentiation into adipocytes [26].

Compared with the L group, the expression of genes related to terminal differentiation (committing white adipocytes to mature white adipocytes) were upregulated in the H group. The *DGAT2*, *CIDEA,* and *SCD* genes are involved in fat synthesis and promote fat deposition. Diacylglycerol acyltransferase (*DGAT*) is a rate-limiting enzyme that controls triglyceride (TG) synthesis in adipocytes [27]. *DGAT* positively correlates with fat deposition and was identified as a candidate adipose deposition gene in pigs [28]. Cell death Inducing DFF45-like Effector (*CIDEA*) induces lipid storage (obesity) [29]. It is activated by agonists of *PPARs*, which then promote fat deposition. *CIDEA* also negatively regulates uncoupling protein 1 (UCP1), thereby reducing energy metabolism and increasing fat accumulation [30, 31]. Stearoyl-CoA desaturase (SCD) promotes fat deposition by promoting the conversion of fatty acids [32]. The SNPs in the *SCD* gene affect the composition of fatty acids; for instance, the T allele at g.2228 T > C increases the content of monounsaturated fatty acids [33]. In summary, the DEGs identified in this study are significant candidate genes that may regulate IMF content. However, further experiments are needed to explore the specific underlying mechanisms.

In this study, compared with the L group, *DGKA* was downregulated in the H group. Diacylglycerol kinase (DGKA) phosphorylates diacylglycerol (DAG) to phosphatidic acid, thus removing DAG [34]. In addition, *DGKA* is necessary for the VEGFA-triggered (vascular endothelial growth factor-A) angiogenic program, and its activation requires Src tyrosine kinase activity [35]. In agreement, DGK-deficient mice showed reduced lipolytic activity in mouse adipocytes and increased fat accumulation in adipocytes [36]. Therefore, the *DGKA* gene may be a candidate gene for fat deposition. In addition, compared with the L group, the *LEPR* (leptin receptor) gene, which is an essential regulator of lipid metabolism, was downregulated in the H group [37]. According to previous studies, leptin increases lipolysis and oxygen consumption in the white fat tissue of muscle [38, 39]. In the present study, the *ITGAL* gene was upregulated in the H group compared with the L group, which is consistent with the DEGs identified in the analyses of cattle adipose tissues with different IMF contents. In cattle with a high IMF content, certain ECM protein components (*ITGA1*, *ITGB1,* and *COL11A2*) were significantly upregulated [40]. Consequently, DEGs related to ECM can also be considered candidate DEGs for the differences in IMF content.

## Conclusion

This study used RNA-seq technology to compare transcriptome differences that determine IMF content. In total, 167 DEGs were identified, revealing new information related to gene networks and metabolic pathways that may play an important role in IMF deposition in Guangling donkeys. In addition, a large number of novel genes and AS events were detected in the *longissimus pectoralis* muscle, which could be further exploited to find the specific roles of candidate genes (SCD, LEPR, CIDEA, DLK1, DGAT2, ITGAL, HMOX1, WNT10B and DGKA) in IMF deposition.

## Materials and methods

### Animal care

Animal welfare and all animal experimental procedures were performed in accordance with ARRIVE guidelines. Animal sample collection and experimental protocols were all approved by the Animal Ethics Committee of Shanxi Agricultural University (Approval No. SXAU-EAW-2019D.AZ.050601). Throughout the study, the animals were reared in the same environment and with the same diet conditions.

### Sample collection

To improve data reliability and reduce individual differences, a total of 30 Guangling donkeys were raised on a commercial donkey farm in Fanshi County, Xinzhou City, Shanxi Province, China, and 6 donkeys with IMF differences and similar ages were selected (age: 2–3 years old, weight: 232–245 kg; female) for use in this study. All Guangling donkeys were reared under the same natural conditions of uncontrolled room temperature and light with unrestricted access to food and water. The *longissimus dorsi* samples at the 13th rib were aseptically and quickly obtained within 30 min of harvest. The collected samples were stored in liquid nitrogen for immediate storage, and long-term storage was carried out at − 80 °C. According to the China National Standard GB5009.6–2016 “Determination of Fat in Foods in National Food Safety Standard”, the IMF content was determined by the Soxhlet method. A Soxhlet extraction apparatus was used to remove fat and dry the ground meat samples for fat extraction. Petroleum ether was used as a solvent. This was recycled and dried for 8 h. Then, it was weighed to obtain the weight of the bottle containing fat. The IMF content was calculated by a formula. The three *longissimus dorsi* samples with the highest IMF contents and the other three with the lowest IMF contents were selected for transcriptome analysis.

### RNA extraction and quantification

Total RNA was extracted from muscle samples using TRIzol reagent (Life Technologies Corp.) following the manufacturer’s instructions. RNA purity, concentration, and integrity were measured using a Nano Photometer spectrophotometer, Qubit 2.0 fluorometer (California Life Technologies, USA), and Agilent 2100 Bioanalyzer (Agilent Technologies, California, USA), respectively.

### Transcriptome library construction and inspection

Oligo (dT) magnetic beads were used to isolate polyA+ mRNA, and the first strand of cDNA was synthesized by reverse transcription. Then, the RNA template was removed to obtain double-stranded RNA. The cDNA library was subjected to AMPure XP bead (Beckman-Coulter, Beverly, USA) purification and PCR enrichment. The constructed library was subjected to preliminary quantification and concentration estimation (effective library concentration > 2 nM) using an Agilent Bioanalyzer 2100, Qubit 2.0 Fluorometer, and Q-PCR. Sequencing (100 bp read) was performed using the Illumina NovaSeq sequencing system (Nebraska, USA).

### Data quality control

Based on the Illumina NovaSeq 6000 platform, transcriptome analysis of 6 samples of the Guangling donkey longissimus dorsi muscles from the H group and L group was carried out. Fastp v 0.19.3 was used to filter the original data, mainly to remove reads with adapters. When the N content in any sequencing reads exceeded 10% of the base number of the reads, the paired reads were removed. When the number of low-quality (Q < =20) bases contained in the reads exceeded 50% of the bases of the reads, the paired reads were removed. Clean reads of 55.79 Gb were obtained after filtering the data, and all subsequent analyses were based on the clean reads. RNA sequencing data have been submitted to the National Center for Biotechnology Information (NCBI) BioProject database (SRA: PRJNA658642).

### Mapping and quantification of gene expression levels

The reference genome (*Equus asinus* ASM130575v1) and its annotation files were downloaded from the NCBI database. HISAT v2.1.0 was used to construct the index and compare clean reads to the reference genome. FeatureCounts v1.6.2 was used to calculate the gene alignment and FPKM. FPKM is currently the most commonly used method to estimate gene expression levels.

### DEG analysis

To avoid the differences caused by the different compositions of the sequencing library, DESeq2 v1.22.1 was used to analyse the differential expression between the two groups, and the *P* value was corrected using the Benjamini & Hochberg method. FeatureCount v1.6.1 [41] computes a count matrix for differential expression analysis. The corrected *P* value and |log2-fold-change| were used as the thresholds for significant differential expression. The differential genetic screen between the high-fat and low-fat donkeys was |log2 Fold-Change| > = 1, and the FDR value was < 0.05 [42, 43].

### Functional annotation and enrichment analysis of DEGs

To explore the relationship between the DEGs and IMF deposition, GO and KEGG pathway enrichment analyses of the DEGs were performed using the DAVID (Database for Annotation, Visualization, and Integrated Discovery) program annotation tools [44].

### Interaction network of the DEGs

GeneMANIA [45] was used to construct the interaction network of the DEGs. The network weight method automatically selected by the system (the default) reflects the contribution and relevance of each gene in the input list. The protein interaction network corresponding to the DEGs was constructed using the STRING database [46]. The analysis results were visualized by Cytoscape ver 3.7.2 [47] for nodes, connecting lines, etc. The cytoHubba plug-in [48] was used to screen key proteins in the interaction network. The number of network nodes was set to 38, and the topology analysis method was maximal clique centrality (MCC).

### qRT–PCR analysis

Seven DEGs were validated by qRT–PCR using the TB Green® Premix Ex Taq™ II (TaKaRa) kit to verify the reliability and accuracy of the sequencing data. Primers, designed by the GenScript Primer Design online tool, were synthesized by Beijing Qingke Xinye Technology (Table 4). The 20 μL reaction system included 10 μL of 2 × SYBR qPCR Mix, 2 μL of cDNA, 0.8 μL of forward and reverse primers (10 μM), 6 μL of ddH_2_O, and 0.4 μL of ROX Reference Dye (50X). The qRT–PCR program was as follows: 95 °C for 1 min; 95 °C for 15 s; 60 °C for 1 min; and a final extension at 72 °C for 20 s, for a total of 40 cycles. Each amplification had three replicates. The relative expression levels of the genes were quantified by the 2^-△△CT^ method; the *β-actin* gene was used as the internal reference gene. The significant differences in gene expression levels between the H (high) and L (low) groups were statistically identified using the t test (GraphPad Prism 7).

**Table 4:** Primer sequences and product sizes

Gene	Primer	Sequences of primers/5′ → 3′	Annealing temperature/°C	Production length/bp
*DGAT2*	Forward	CTGCCCTACCCGAAGCCTAT	60.0	98
	Reverse	GCGTGGTACAGGTCGATGTC		
*SCD*	Forward	TGTCGTGTTGCTGTGCTTCA	60.0	111
	Reverse	AGCACAAGAGCGTAACGCAA		
*LEPR*	Forward	ATCGGAAGAGTGGCCTCTGG	60.0	118
	Reverse	GTGGTCGAGTCTGGTTGCTG		
*DLK1*	Forward	CACCATGGGCATCGTCTTCC	60.0	179
	Reverse	CACCAGCCTCCTTGCTGAAG		
*WNT10B*	Forward	CGGTTTCCGTGAGAGTGCTT	60.0	124
	Reverse	CTCACCACTGCCCTTCCAGT		
*CIDEA*	Forward	CCAGCAGCCAAAGAGATCGG	60.0	101
	Reverse	ACATGGTGGCCTTCACGTTG		
*DGKA*	Forward	CTGGACAGCTCAGAAGTGGA	60.0	150
	Reverse	CTCAGCTAGGGAGACAGAGC		
*β-actin*	Forward	CGACATCCGTAAGGACCTGT	60.0	100
	Reverse	CAGGGCTGTGATCTCCTTCT		

### Novel gene analysis and prediction of alternative splicing

StringTie v1.3.4d was used for novel gene prediction. StringTie applies network streaming algorithms to identify de novo splice transcripts. Compared with Cufflinks and other software, StringTie can splice a more complete and accurate transcript, and the splicing speed is faster. CPC2, PLEK and CNCI software were used to jointly predict gene coding potential. If the three crossover results showed “Non-Coding”, it indicated that the predicted genes or transcript had no coding ability. Blastx was used to align the novel genes with KEGG (Kyoto Encyclopedia of Genes and Genomes), GO (Gene Ontology), NR (NCBI nonredundant protein sequences), Swiss-Prot (a manually annotated and reviewed protein sequence database), trEMBL (Translation of EMBL), and KOG (Eukaryotic Orthologous Groups of proteins) databases, setting the e-value as 1e-5. The Pfam database uses HMMER software to perform the alignment and extract all annotation results.

AS (alternative splicing) events were identified by rMATS v4.0.2 [49] software and validated with the Junction County (JC) method. Five different splicing patterns were detected: (A) SE: skipped exon; (B) A5SS: alternative 5′ splice site; (C) A3SS: alternative 3′ splice site; (D) MXE: mutually exclusive exons, and (E) RI: retained intron.

## Supplementary Information

**Additional file 1: Table S1.** Gene names/information about different genes screend.

**Additional file 2: Table S2.** Differential genes GO functional enrichment analysis results.

**Additional file 3: Table S3.** 38 differential genes related to fat metabolism.

**Additional file 4: Table S4.** Details sheet for novel genes.

## Data Availability

The RNA sequencing data in this study can be obtained from the National Center for Biotechnology Information (NCBI) (https://www.ncbi.nlm.nih.gov/bioproject/?term=PRJNA658642), registration number PRJNA658642.
